# *Leaving no one behind?* An equity analysis of the HIV care cascade among a cohort of people living with HIV in Manitoba, Canada

**DOI:** 10.1186/s12889-021-10225-w

**Published:** 2021-02-04

**Authors:** Leigh M. McClarty, James F. Blanchard, Marissa L. Becker

**Affiliations:** Institute for Global Public Health, Rady Faculty of Health Sciences, University of Manitoba, R065 Medical Rehabilitation Building, 771 McDermot Ave, Winnipeg, MB R3E 0T6 Canada

**Keywords:** HIV, Manitoba, Cohort studies, Epidemiology, Retention in care, Patient care

## Abstract

**Background:**

Manitoba is a central Canadian province with annual rates of new HIV infections consistently higher than the Canadian average. National surveillance statistics and data from the provincial HIV care program suggest that epidemiological heterogeneity exists across Manitoba. New HIV cases are disproportionately reported among females, Indigenous-identifying individuals, and those with a history of injection drug use. Given the heterogeneity in acquisition, it is of interest to understand whether this translates into inequalities in HIV care across Manitoba.

**Methods:**

A sample of 703 participants from a clinical cohort of people living with HIV in Manitoba, with data current to the end of 2017, was used to conduct cross-sectional, disaggregated analyses of the HIV care cascade to identify heterogeneity in service coverage and clinical outcomes among different groups receiving HIV care in Manitoba. Equiplots are used to identify and visualize inequalities across the cascade. Exploratory multivariable logistic regression models quantify associations between equity variables (age, sex, geography, ethnicity, immigration status, exposure category) and progression along the cascade. Adjusted odds ratios (AOR) and 95% confidence intervals (95%CI) are reported.

**Results:**

Equity analyses highlight inequalities in engagement in and coverage of HIV-related health services among cohort participants. Equiplots illustrate that the proportion of participants in each cascade step is greater for those who are older, white, non-immigrants, and report no history of injection drug use. Compared to those living in Winnipeg, participants in eastern Manitoba have greater odds of achieving virologic suppression (AOR[95%CI] = 3.8[1.3–11.2]). The odds of Indigenous participants being virologically suppressed is half that of white participants (AOR[95%CI] = 0.5[0.3–0.7]), whereas African/Caribbean/Black participants are significantly less likely than white participants to be *in care* and *retained in care* (AOR[95%CI] = 0.3[0.2–0.7] and 0.4[0.2–0.9], respectively).

**Conclusions:**

Inequalities exist across the cascade for different groups of Manitobans living with HIV; equiplots are an innovative method for visualizing these inequalities. Alongside future research aiming to understand *why* inequalities exist across the cascade in Manitoba, our equity analyses can generate hypotheses and provide evidence to inform patient-centred care plans that meet the needs of diverse client subgroups and advocate for policy changes that facilitate more equitable HIV care across the province.

## Background

Manitoba is a central Canadian province where annual rates of new HIV infections are consistently higher than the national average (7.9 vs. 6.9 per 100,000 population, respectively, in 2018) [1]. Injection drug use (33.9%), condomless anal sex between men (24.4%), and condomless vaginal (heterosexual) sex (20.9%) are the most commonly identified HIV risk exposures in Manitoba [2], and new infections in 2018 were disproportionately high, compared to the rest of Canada, among individuals identifying as Indigenous (50% vs. 19.3%) and female (40% vs. 29.3%) [1, 3]. Additionally, notable heterogeneity in rates of new HIV infection exists across the province by geography, age, and sex [3]—in 2018, 77.6% of new diagnoses occurred in Winnipeg, the provincial capital and main urban centre, and among newly diagnosed females, 11.6% were ≤ 19 years (compared to 1.6% of males) and 14.0% were ≥ 60 years (compared to 3.1% of males) [3]. At the end of 2018, Manitoba Health, Seniors and Active Living (MSHAL), estimated that 1572 people were living with HIV in the province (personal communication, J. Paul, April 16, 2020), and the Manitoba HIV Program—the primary provider of HIV care in the province—estimated that approximately 1400 people were in care in the same year [2].

Over the past decade of HIV research, heavy emphasis has been placed on the HIV care cascade (“the cascade”)—a framework and analytic tool providing insights into the continuum of care services for people living with HIV [4–6]—and its simplified counterparts the 90–90-90 Initiative [7]. Conventionally, cascades use aggregate data to illustrate the proportion of individuals in a population of people living with HIV who have been diagnosed, linked to HIV care services, retained in care, then initiated and sustained on HIV treatment to, ultimately, reach virologic suppression. Using aggregate data to illustrate the continuum of HIV care for an entire population is useful insofar as it can provide a general picture of points of “leakage” or “bottlenecks” within a health system or care program. However, relying on aggregate data to paint a picture of an entire population risks obscuring the underlying heterogeneity among and between individuals and groups who make up the population. To generate evidence that can help to inform the development and optimization of interventions and programs addressing inequities in HIV care, it is crucial to conduct equity analyses that generate disaggregated cascades to showcase nuances and highlight inequalities across the cascade steps within a population.

In 2015, all 193 Member States of the United Nations agreed upon the 2030 Agenda for Sustainable Development, comprising seventeen Sustainable Development Goals (SDGs), which built upon the Millennium Development Goals (MDGs) introduced fifteen years earlier [8]. At the core of the SDGs is the notion of *leaving no one behind*, which, “represents the unequivocal commitment … to reduce the inequalities and vulnerabilities that leave people behind and undermine the potential of individuals and of humanity as a whole” [9 , p. 6]. This idea underscores the interconnectedness of the SDGs and principles of health equity [10]—a noted limitation of the MDGs [11]. As such, under the auspices of the SDGs [12], there is a need for research that focuses on identifying (health) inequalities that exist, examining the factors that perpetuate and exacerbate these inequalities, understanding how specific inequalities are related to broader health inequity [13], and developing strategies to minimize or, ideally, eliminate them. As noted in SDG 17, to adequately assess (in)equities, it is necessary that data are disaggregated by socioeconomic, demographic and other relevant, context-specific characteristics [11, 12].

Publicly available HIV epidemiological data in Manitoba are limited to reports published by the Public Health Agency of Canada (PHAC) [1] and MHSAL [3], which focus solely on surveillance data. As such, local understandings of inequalities in HIV care and clinical outcomes among different groups in the province are rudimentary. In 2013—through the support of a multi-site program of research, *Advancing Primary Healthcare for Persons Living with HIV in Canada* (the LHIV Study), funded by the Canadian Institutes of Health Research [14]—a prospective clinical cohort of people living with HIV and/or receiving HIV care in Manitoba was established as an embedded research project within the Manitoba HIV Program [15]. The establishment of the LHIV-Manitoba cohort opens up numerous analytic opportunities to better understand HIV epidemiology in Manitoba and, for the first time, provides access to de-identified, individual-level clinical data, allowing for disaggregated analyses to take place.

Here, building upon previous work [15–17], we use equiplots to present disaggregated cascade analyses (by age, sex, geography, ethnicity, immigration status, and HIV exposure category) that visualize inequalities in service uptake and clinical outcomes among LHIV-Manitoba cohort participants who were alive as of 31 December 2017 and had received an HIV diagnosis on or before that date. Exploratory multivariable logistic regression analyses are used to quantify these inequalities, generate hypotheses, and provide guidance for future cascade research in Manitoba. In conjunction with future research to understand *why* identified inequalities exist across the cascade [6] and how these inequalities contribute to health inequities [13], our examination of the cascade through an equity lens [18, 19] will provide Manitoba’s provincial care program with evidence needed to develop patient-centred care plans that meet the needs of heterogeneous client subgroups, and to advocate for policy changes addressing inequities in HIV care across the province.

## Methods

### Study setting

Manitoba has a population of 1.36-million people, spanning over 550,000 km^2^. Approximately 57% of Manitoba’s population lives in the capital city of Winnipeg, 37% in the western, eastern, and southern regions (rural), and 6% in the north (rural-remote) [20]. HIV care in Manitoba is primarily provided through the Manitoba HIV Program, comprising three clinics—two in Winnipeg and one in a smaller, rural city in southwestern Manitoba. As such, the majority of Program clients living in rural and rural-remote regions of the province are required to travel substantial distances to attend clinic appointments. The Manitoba HIV Program employs a multidisciplinary care model encompassing a full complement of health and social service providers, including HIV specialists and family physicians, nurses, pharmacists, social workers, and other allied health professionals. Despite Canada’s publicly funded healthcare system, no Canadian provinces or territories have a single drug plan that provides universal coverage for all, and out-of-pocket expenses associated with prescription medications vary substantially depending on an individual’s insurance coverage [21].

### Data sources

Data used to generate disaggregated, cross-sectional analyses of the Manitoban cascade are derived from the LHIV-Manitoba clinical cohort dataset, comprising individual-level, de-identified clinical data linked to provincial administrative health databases. Clinical cohort data are linked to the most recently available administrative health datasets and are reported up to 31 December 2017. Manitoba’s administrative datasets include individual-level records for nearly all contacts within the province’s healthcare system. Linked administrative data within the cohort include variables pertaining to physician visits, hospital admissions, prescription drug dispensation, and laboratory testing, including quantitative HIV plasma viral load data [15]. The process of establishing the cohort, a complete profile of cohort participants, and a comprehensive description of the datasets is detailed in earlier work [15]. Briefly, the cohort includes 890 unique individuals, comprising 63.6% of the estimated 1400 clients receiving care from the Manitoba HIV Program and 56.6% of the estimated 1572 people living with diagnosed HIV in the province. All adults (≥18 years) living with HIV and/or receiving HIV care in Manitoba were eligible for participation, unless under the jurisdiction of the Public Guardian and Trustee of Manitoba or otherwise unable to make their own healthcare decisions. Potential participants have the opportunity to agree to any combination of three separate components of the LHIV-Manitoba cohort study: (i) have their clinical data collected; (ii) have their clinical data linked to provincial administrative health datasets; and/or (iii) indicate interest in being approached about participating in future HIV research studies [15]. In total, 725 (81.5%) cohort participants agreed to have their data extracted from clinical records, and 703 (78.9%) of those participants also agreed to have their clinical data anonymously linked to administrative health databases. A limited clinical dataset exists for an additional 165 individuals whose clinical records were reviewed posthumously and linked to administrative health databases. In total, the linked dataset comprises 868 participants. Missing data were minimal throughout the cohort dataset—9.1% (*n* = 81) of all participants who were alive at enrolment are missing observations from at least one key variable of interest [16]. Missing data points are excluded from relevant analyses. All cohort participants who were alive as of 31 December 2017, had received a positive HIV diagnosis at any point on or before the same date, and had provided written, informed consent to have their clinical data reviewed and linked to administrative health datasets were included in a baseline HIV care cascade model and subsequent equity analyses.

### Equity analyses

An HIV care cascade has previously been developed and specifically tailored to accommodate available data sources within Manitoba [16]. The existence of a single insurer responsible for payment of the majority of health services in the province (MHSAL) and linkable, population-based administrative health databases afforded opportunities for developing sensitive and data-intensive indicator definitions [16, 17] similar to definitions used in other provinces [22, 23]. Table 1 outlines the established definitions for five indicators representing each step of the Manitoban cascade, which were derived data from both clinical cohort data (CD4 cell count data) and linked administrative health datasets (viral load, physician visits, and drug dispensation data).

**Table 1:** Indicator definitions for the Manitoban HIV care cascade model

CASCADE STEP	DEFINITION
***Alive and diagnosed***	Cohort participants who were alive as of 31 December 2017 and received a positive diagnosis for HIV at any point on or before 31 December 2017.
***In care***	Among those *alive and diagnosed*: Cohort participants who had at least 1 viral load test or CD4 count or physician visit for HIV^*^ within the first 180 days of 2017 (or within 180 days of HIV diagnosis, if diagnosed in 2017).
***Retained in care***	Among those *in care*: Cohort participants who had at least 2 occurrences of a viral load test and/or a physician visit for HIV, at least 90 days apart, in 2017.
***On treatment***	Among those *retained in care*: Cohort participants who had at least 2 antiretroviral drug dispensations, at least 90 days apart, in 2017.
***Virologically suppressed***	Among those *on treatment*: Cohort participants whose last viral load test result in 2017 was below 200 HIV RNA copies/mL***.***

^*^ Physician visits were identified using medical claims (captured through physician billings) associated with HIV-related International Classifications of Disease (ICD)-9 and/or ICD-10 codes

Throughout the paper, the term “inequality” describes measured differences in outcomes between groups, whereas “inequities” refer to the implications of population-level inequalities, which, in the context of public health, are understood to be fundamentally unjust or unfair [13]. To examine inequalities across the Manitoban care cascade, we identified relevant equity variables available within the cohort by which each cascade step indicator was then disaggregated. These variables—which have been recommended for use in equity analyses previously [10, 11, 19]—included age; sex; geographic location of residence; self-identified ethnicity; immigration status; and primary HIV exposure category, identified using a “risk hierarchy” framework [24]. A participant’s geography is categorized by provincial Regional Health Authority (Supplementary Figure, see Additional File 1), which is inferred from the postal code of residence recorded in the cohort database [25]. Participants are categorized as “immigrants” if they were foreign-born and had immigrated to Canada in 2001 or later [26] (Maritim C, McClarty L, Leung S, Bruce S, Restall G, Migliardi P, Becker M. HIV treatment outcomes among newcomers living with HIV in Manitoba, Canada JAMMI In press), or “non-immigrants” if they were either Canadian-born or foreign-born, having immigrated to Canada before 2001.

#### Equiplots

To aid in the visualization of equity analyses, equiplots [27] were used to present the proportion of participants within each group—defined by key equity variables—who reach each cascade step, thus illustrating inequalities in progression along the cascade. Data were excluded from equiplot analyses for participants living outside of Manitoba or without a known permanent address. Equiplots were generated in Stata 15.1 (College Station, TX) using code available through the International Centre for Equity in Health, Universidad Federal de Pelotas, Brazil [27].

#### Multivariable logistic regression models

The *in care*, *retained in care*, *on treatment*, and *virologically suppressed* cascade steps (as defined in Table 1) were used as dichotomous outcome variables and the aforementioned equity variables were included as categorical exposure variables in multivariable logistic regression models. For each of the four outcomes of interest, six individual models were constructed to quantify associations between individual equity variables and reaching each cascade step. All equity variables were converted into dummy variables in the models and were tested for collinearity prior to inclusion. Cohort participants living outside of Manitoba and those who had no known address recorded in their medical files were excluded from multivariable models quantifying inequalities across the cascade, disaggregated by geographic region (Model 3 in Table 4). Adjusted odds ratios (AOR) are presented using 95% confidence intervals (95%CI) to assess statistical significance. Crude analyses from unadjusted models are presented in the Supplementary Table (see Additional File 2). All statistical analyses were performed using Stata 15.1 (College Station, TX).

## Results

In total, 703 cohort participants were alive and had received an HIV diagnosis as of 31 December 2017. Distributions of key equity variables among these participants, disaggregated by cascade step, are presented in Table 2.

**Table 2:** Distribution of equity variables among LHIV-Manitoba cohort participants in each step of the HIV care cascade as of 31 December 2017

HIV care cascade step	Alive and diagnosed		In Care		Retained in care		On treatment		Virologically suppressed	
Total participants in cascade step, *N*	703		638		606		573		523	
	***n***	**%**	***n***	**%**	***n***	**%**	***n***	**%**	***n***	**%**
**Age range, in years**										
18–29	41	5.8	32	5.0	27	4.5	24	4.2	21	4.0
30–39	127	18.1	109	17.1	105	17.3	94	16.4	79	15.1
40–49	200	28.5	180	28.2	173	28.6	164	28.6	149	28.5
50–59	233	33.1	220	34.5	206	34.0	201	35.1	188	36.0
60+	102	14.5	97	15.2	95	15.7	90	15.7	86	16.4
Mean (SD)	48.5 (11.5)		49.1 (11.3)		49.3 (11.3)		49.5 (11.2)		49.9 (11.2)	
Median (IQR)	49.3 (40.3–55.9)		49.9 (40.9–56.1)		49.9 (40.9–56.4)		50.2 (41.3–56.4)		50.6 (42.3–56.7)	
**Sex**										
Male	507	72.1	463	72.6	438	72.3	414	72.3	380	72.7
Female	196	27.9	175	27.4	168	27.7	159	27.8	143	27.3
**Geography, by region**										
Winnipeg	572	81.4	523	82.0	495	81.7	467	81.5	427	81.6
Northern Manitoba	25	3.6	22	3.5	22	3.6	21	3.7	18	3.4
Western Manitoba	25	3.6	19	3.0	18	3.0	18	3.1	18	3.4
Eastern Manitoba	44	6.3	43	6.7	41	6.8	40	7.0	40	7.7
Southern Manitoba	27	3.8	24	3.8	23	3.8	21	3.7	15	2.9
Other ^a^	10	1.4	7	1.1	7	1.2	6	1.1	5	1.0
**Self-identified ethnicity**										
White	307	43.7	289	45.3	279	46.0	266	46.4	252	48.2
Indigenous	269	38.3	243	38.1	225	37.1	207	36.1	175	33.5
Sub-Saharan African/Caribbean/Black	91	12.9	76	11.9	74	12.2	72	12.6	70	13.4
Other ^b^	36	5.1	30	4.7	28	4.6	28	4.9	26	5.0
**Immigration status** ^c^										
Non-immigrant	613	87.2	565	88.6	536	88.5	505	88.1	457	87.4
Immigrant	90	12.8	73	11.4	70	11.6	68	11.9	66	12.6
**Self-identified HIV exposure category**										
Condomless anal sex between men (MSM) only	243	34.6	224	35.1	214	35.3	199	34.7	191	36.5
MSM + injection drug use (IDU)	20	2.8	18	2.8	17	2.8	16	2.8	16	3.1
IDU only	110	15.7	99	15.5	91	15.0	85	14.9	68	13.0
Condomless vaginal (heterosexual) sex	310	44.1	281	44.0	268	44.2	257	44.9	232	44.4
No identified risk/Other risk ^d^	20	2.8	16	2.5	16	2.6	16	2.8	16	3.1

^a^ Includes participants with no known address and those with permanent addresses outside of Manitoba. These participants are excluded from equiplot and multivariable logistic regression analyses

^b^ Includes Latin American, East/Southeast Asian, South Asian, West Asian/North African/Middle Eastern

^c^ “Immigrants” are foreign-born participants who immigrated to Canada in 2001 or later [26]. “Non-immigrants” are either Canadian-born participants or foreign-born participants who immigrated to Canada before 2001

^d^ “Other risk” includes recipients of blood/blood products, perinatal acquisition, occupational exposure

**Fig. 1:**
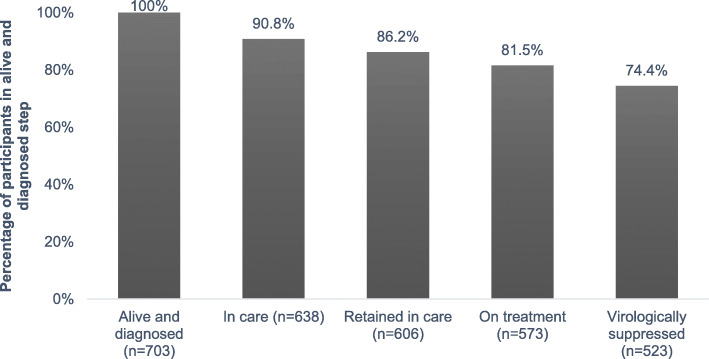
Baseline HIV care cascade model for Manitoba. *Alive and diagnosed* step is denominator for each subsequent cascade step

Among the 703 *alive and diagnosed* participants (100%), 638 (90.8%) met the definition of *in care*, 606 (86.2%) were *retained in care*, 573 (81.5%) were *on treatment*, and 523 (74.4%) had reached the *virologically suppressed* step of the cascade at the end of the 2017 calendar year (Fig. 1).

### Examining the cascade through an equity lens

Analyzing the Manitoba cascade data through an equity lens highlights a number of inequalities in the proportion of cohort participants who reached the *in care*, *retained in care*, *on treatment*, and *virologically suppressed* steps. Data corresponding to each of the presented equiplots are presented in Table 3.

**Table 3:** Number and percentage of participants in each HIV care cascade step as a percentage of those *alive and diagnosed* as of 31 December 2017, disaggregated by equity variables

HIV care cascade step	Alive and diagnosed		In Care		Retained in care		On treatment		Virologically suppressed	
Total participants in cascade step, *N*	703		638		606		573		523	
	*n*	% of *alive and diagnosed*	*n*	% of *alive and diagnosed*	*n*	% of *alive and diagnosed*	*n*	% of *alive and diagnosed*	*n*	% of *alive and diagnosed*
**Age range, in years**										
18–29	41	100	32	78.0	27	65.9	24	58.5	21	51.2
30–39	127	100	109	85.8	105	82.7	94	74.0	79	62.2
40–49	200	100	180	90.0	173	86.5	164	82.0	149	74.5
50–59	233	100	220	94.4	206	88.4	201	86.3	188	80.7
60+	102	100	97	95.1	95	93.1	90	88.2	86	84.3
**Sex**										
Male	507	100	463	91.3	438	86.4	414	81.7	380	75.0
Female	196	100	175	89.3	168	85.7	159	81.1	143	73.0
**Geography, by region** ^a^										
Winnipeg	572	100	523	91.4	495	86.5	467	81.6	427	74.7
Northern Manitoba	25	100	22	88.0	22	88.0	21	84.0	18	72.0
Western Manitoba	25	100	19	76.0	18	72.0	18	72.0	18	72.0
Eastern Manitoba	44	100	43	97.7	41	93.2	40	90.9	40	90.9
Southern Manitoba	27	100	24	88.9	23	85.2	21	77.8	15	55.6
**Self-identified ethnicity**										
White	307	100	289	94.1	279	90.9	266	86.6	252	82.1
Indigenous	269	100	243	90.3	225	83.6	207	77.0	175	65.1
Sub-Saharan African/Caribbean/Black	91	100	76	83.5	74	81.3	72	79.1	70	76.9
Other ^b^	36	100	30	83.3	28	77.8	28	77.8	26	72.2
**Immigration status** ^c^										
Non-immigrant	613	100	565	92.2	536	87.4	505	82.4	457	74.6
Immigrant	90	100	73	81.1	70	77.8	68	75.6	66	73.3
**Self-identified HIV exposure category**										
Condomless anal sex between males (MSM) only	243	100	224	92.2	214	88.1	199	81.9	191	78.6
MSM + injection drug use (IDU)	20	100	18	90.0	17	85.0	16	80.0	16	80.0
IDU only	110	100	99	90.0	91	82.7	85	77.3	68	61.8
Condomless vaginal (heterosexual) sex	310	100	281	90.6	268	86.5	257	82.9	232	74.8
No identified risk/Other risk ^d^	20	100	16	80.0	16	80.0	16	80.0	16	80.0

^a^ *N* = 693 in the *alive and diagnosed* step. Data from 10 participants who either lived outside of Manitoba or lacked current address data in their medical records were removed for equiplot and multivariable logistic regression analyses

^b^ Includes Latin American, East/Southeast Asian, South Asian, West Asian/North African/Middle Eastern

^c^ “Immigrants” are foreign-born participants who immigrated to Canada in 2001 or later [26]. “Non-immigrants” are either Canadian-born participants or foreign-born participants who immigrated to Canada before 2001

^d^ “Other risk” includes recipients of blood/blood products, perinatal acquisition, occupational exposure

#### Age

**Fig. 2:**
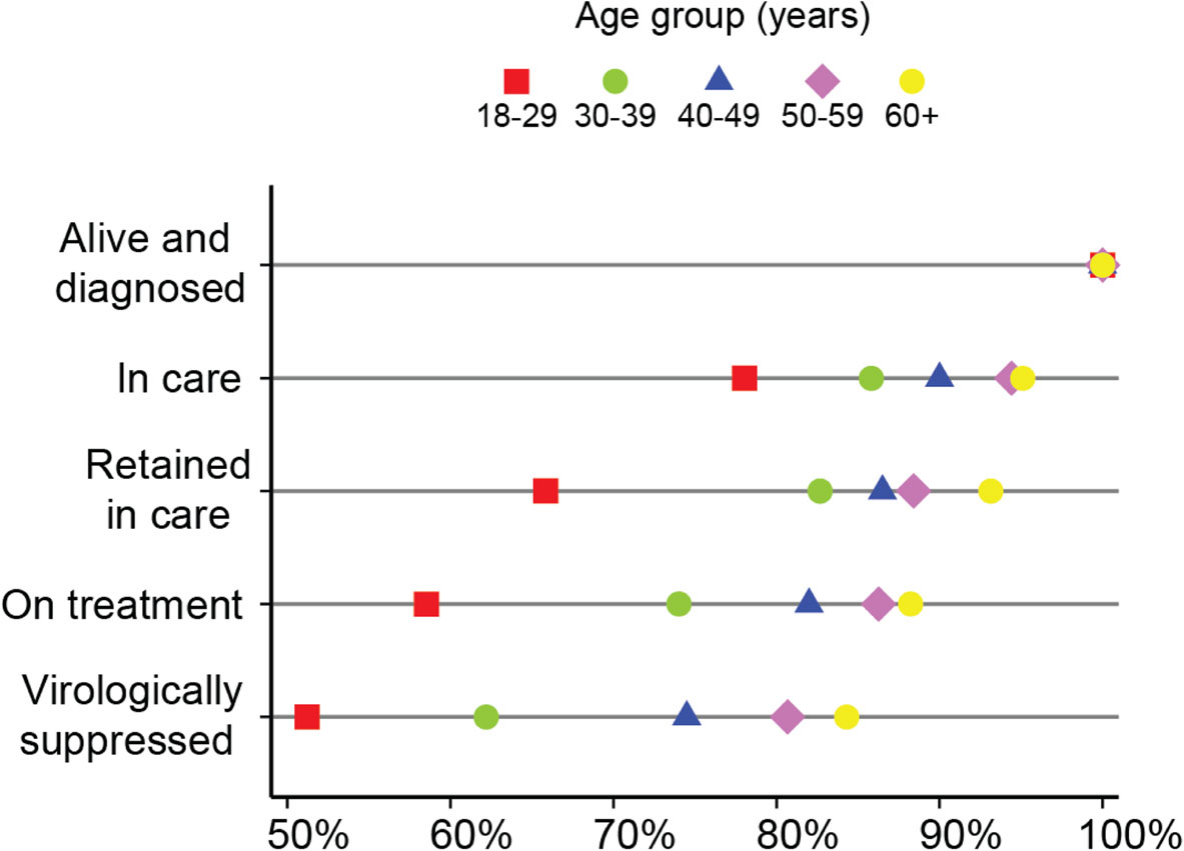
Inequalities across the Manitoban HIV care cascade, by age group. *N* = 703 at *alive and diagnosed*

Disaggregated by age group, cascade data (Fig. 2 and Table 3) indicate that a greater proportion of participants reach all four cascade steps with each increase in age group; multivariable logistic regression analyses (Model 1 in Table 4) support this visual interpretation. Compared to participants aged 18–29 years, those in the 40–49, 50–59, and 60–69 year age groups have significantly greater odds of being *in care* (AOR[95%CI] = 2.51[1.03–6.11], 4.53[1.73–11.89], 5.05[1.52–16.69], respectively), *retained in care* (AOR[95%CI] = 3.28[1.51–7.13], 3.69[1.67–8.14], and 6.37[2.27–17.85], respectively), *on treatment* (AOR[95%CI] = 2.01[0.96–4.24], 3.27[1.58–6.77], 4.39[2.08–9.30], 4.99[2.05–12.15], respectively), and *virologically suppressed* (AOR[95%CI] = 2.77[1.37–5.59], 3.75[1.83–7.68], 4.53[1.96–10.44], respectively). However, participants aged 30–39 years are only at significantly greater odds of being *retained in care* compared to the 18–29 year reference age group (AOR = 2.39, 95%CI:1.07–5.34).

**Table 4:** Adjusted odds ratios (AOR) and 95% confidence intervals (95%CI) estimated using multivariable logistic regression models to quantify associations between equity variables (age, sex, ethnicity, immigration status, geography, HIV exposure categories) and reaching each step of the HIV care cascade among LHIV-Manitoba clinical cohort participants as of 31 December 2017

HIV CARE CASCADE STEP	IN CARE		RETAINED IN CARE		ON TREATMENT		VIROLOGICALLY SUPPRESSED	
Total participants in cascade step, *N*	638		606		573		523	
	**AOR**	**95%CI**	**AOR**	**95%CI**	**AOR**	**95%CI**	**AOR**	**95%CI**
**Age range, in years** (Model 1) ^a^								
18–29	*Ref.*	–	*Ref.*	–	*Ref.*	–	*Ref.*	–
30–39	1.62	0.65–4.02	2.39	1.07–5.34	2.01	0.96–4.24	1.57	0.77–3.24
40–49	2.51	1.03–6.11	3.28	1.51–7.13	3.27	1.58–6.77	2.77	1.37–5.59
50–59	4.53	1.73–11.89	3.69	1.67–8.14	4.39	2.08–9.30	3.75	1.83–7.68
60+	5.04	1.52–16.69	6.37	2.27–17.85	4.99	2.05–12.15	4.53	1.96–10.44
**Sex** (Model 2) ^b^								
Male	*Ref.*	–	*Ref.*	–	*Ref.*	–	*Ref.*	–
Female	1.29	0.70–2.38	1.43	0.84–2.42	1.51	0.94–2.42	1.45	0.95–2.22
**Geography, by region** (Model 3) ^c^								
Winnipeg	*Ref.*	–	*Ref.*	–	*Ref.*	–	*Ref.*	–
Northern Manitoba	0.64	0.17–2.35	1.23	0.35–4.41	1.37	0.44–4.26	1.06	0.42–2.70
Western Manitoba	0.33	0.12–0.90	0.44	0.17–1.11	0.65	0.26–1.66	1.03	0.41–2.61
Eastern Manitoba	3.04	0.40–23.04	1.91	0.56–6.48	2.27	0.77–6.64	3.82	1.31–11.17
Southern Manitoba	0.64	0.18–2.35	0.95	0.31–2.95	0.92	0.35–2.45	0.55	0.24–1.27
**Self-identified ethnicity** (Model 4) ^d^								
White	*Ref.*	–	*Ref.*	–	*Ref.*	–	*Ref.*	–
Indigenous	0.72	0.37–1.40	0.57	0.33–0.97	0.57	0.36–0.91	0.45	0.30–0.69
Sub-Saharan African/Caribbean/Black	0.34	0.15–0.73	0.43	0.22–0.86	0.57	0.30–1.08	0.73	0.40–1.33
Other ^e^	0.40	0.14–1.13	0.42	0.17–1.05	0.68	0.28–1.66	0.70	0.31–1.57
**Immigration status** ^f^ (Model 5) ^g^								
Non-immigrant	*Ref.*	–	*Ref.*	–	*Ref.*	–	*Ref.*	–
Immigrant	0.73	0.23–2.33	0.66	0.23–1.86	0.65	0.24–1.77	0.84	0.34–21.0
**Self-identified HIV exposure category** (Model 6)								
Condomless anal sex between males (MSM) only	*Ref.*	–	*Ref.*	–	*Ref.*	–	*Ref.*	–
MSM + injection drug use (IDU)	0.87	0.18–4.19	0.91	0.24–3.43	1.16	0.35–3.79	1.47	0.45–4.78
IDU only	0.79	0.32–1.98	0.66	0.32–1.38	0.87	0.45–1.66	0.48	0.27–0.85
Condomless vaginal (heterosexual) sex	1.11	0.50–2.44	0.96	0.50–1.83	1.29	0.73–2.28	0.84	0.51–1.39
No identified risk/Other risk ^h^	0.41	0.11–1.59	0.63	0.18–2.28	1.05	0.30–3.67	1.08	0.32–3.64

a Multivariable model 1: *n* = 703; adjusted for sex and ethnicity

b Multivariable model 2: *n* = 703; adjusted for age group and ethnicity

c Multivariable model 3: *n* = 693; adjusted for age group, sex, and ethnicity

d Multivariable model 4: *n* = 703; adjusted for age group and sex

^e^ Includes Latin American, East/Southeast Asian, South Asian, West Asian/North African/Middle Eastern

^f^ “Immigrants” are foreign-born participants who immigrated to Canada in 2001 or later [26]. “Non-immigrants” are either Canadian-born participants or foreign-born participants who immigrated to Canada before 2001

g Multivariable models 5 and 6: *n* = 703; adjusted for age group, sex, and ethnicity

^h^ “Other risk” includes recipients of blood/blood products, perinatal acquisition, occupational exposure

#### Sex

**Fig. 3:**
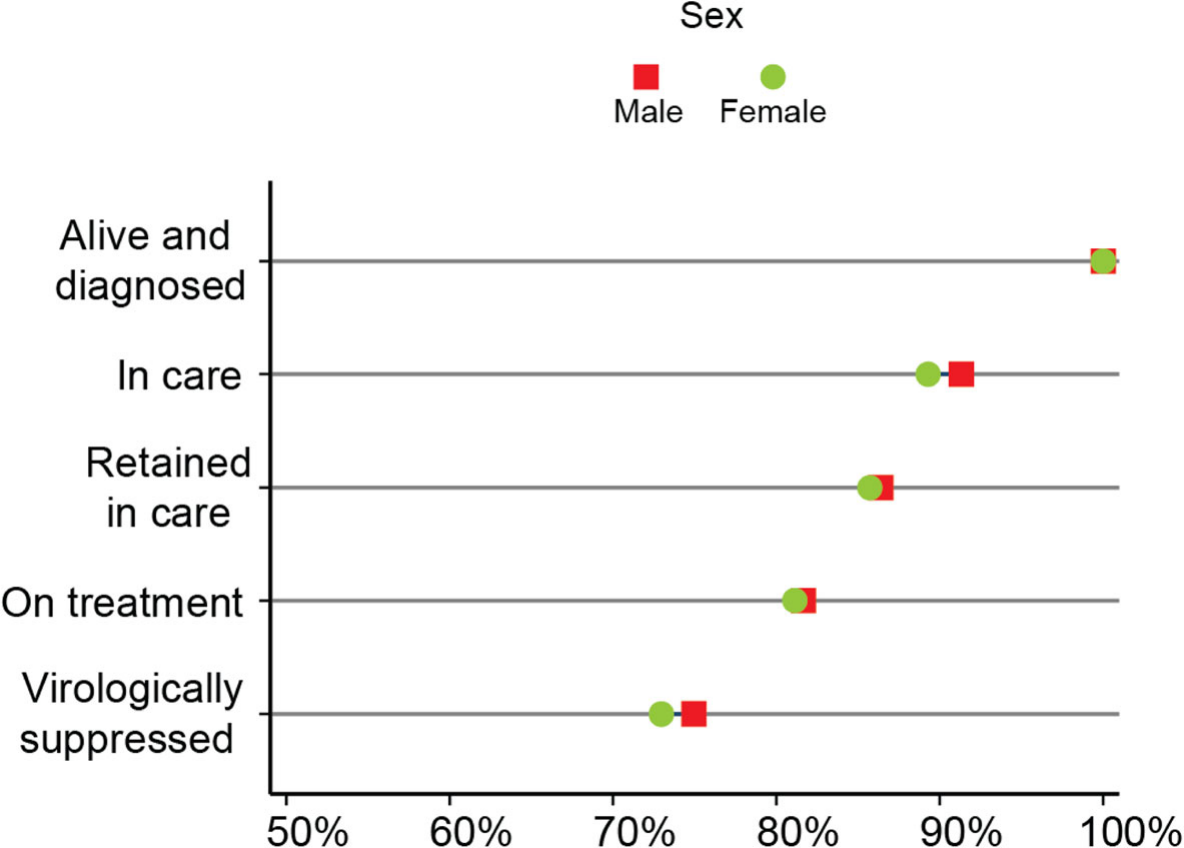
Inequalities across the Manitoban HIV care cascade, by sex. *N* = 703 at *alive and diagnosed*

The equiplot illustrating disaggregated cascade estimates for male and female participants (Fig. 3) highlights similarities between groups at each step with distinguishable, though small, differences in the *in care* (91.3 and 89.3%, respectively) and *virologically suppressed* (75.0 and 73.0%, respectively) cascade steps (Table 3). Data presented in Table 4 (Model 2) indicates that female participants tend to have greater odds of being in each successive cascade step compared to male participants, but these differences are not statistically significant.

#### Geography

**Fig. 4:**
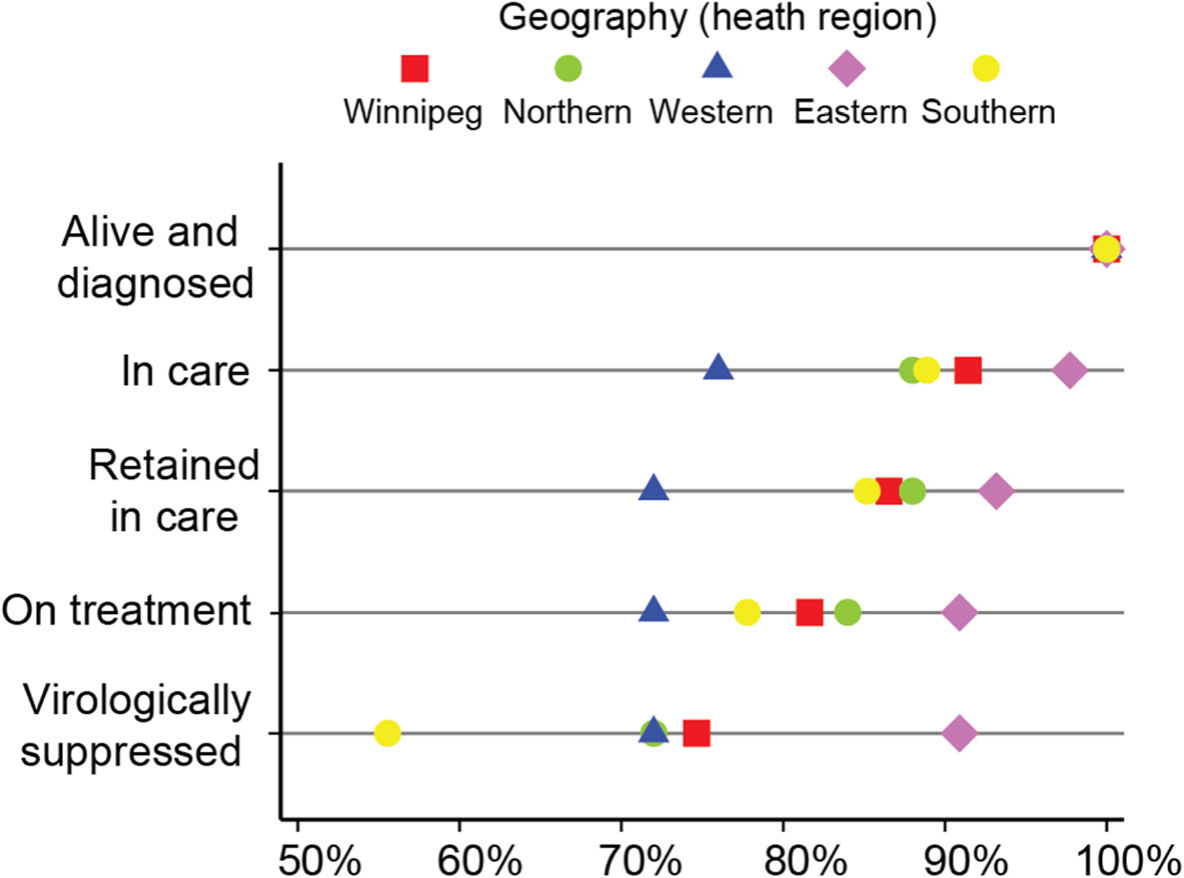
Inequalities across the Manitoban HIV care cascade, by geography. *N* = 693 at *alive and diagnosed*

Inequalities are observed between the proportions of cohort participants in each cascade step when data are disaggregated by geographic region (Fig. 4). Given that the majority (81.9%) of cohort participants reside in the Winnipeg, the proportion of Winnipeg-based cohort participants in each cascade step are similar to the cascade estimates for the entire cohort, depicted in Fig. 1. Visual analysis of equiplot data suggests very little leakage occurs between cascade steps among participants living in eastern Manitoba. However, Fig. 4 and Table 3 highlight substantial drop-off between the *alive and diagnosed* and *in care* steps among western Manitobans (100 to 76.0%), and between the *on treatment* and *virologically suppressed* steps among those living in northern (84.0 to 72.0%) and southern regions (77.8 to 55.6%). Logistic regression Model 3 (see Table 4) indicates that compared to those living in Winnipeg, the odds of being *in care* are significantly lower for participants living in western Manitoba (AOR = 0.33, 95%CI:0.12–0.90). Furthermore, compared to participants living in Winnipeg, the odds of being *virologically suppressed* for those living in eastern Manitoba is nearly four-times greater (AOR = 3.82, 95%CI:1.31–11.17).

#### Ethnicity

**Fig. 5:**
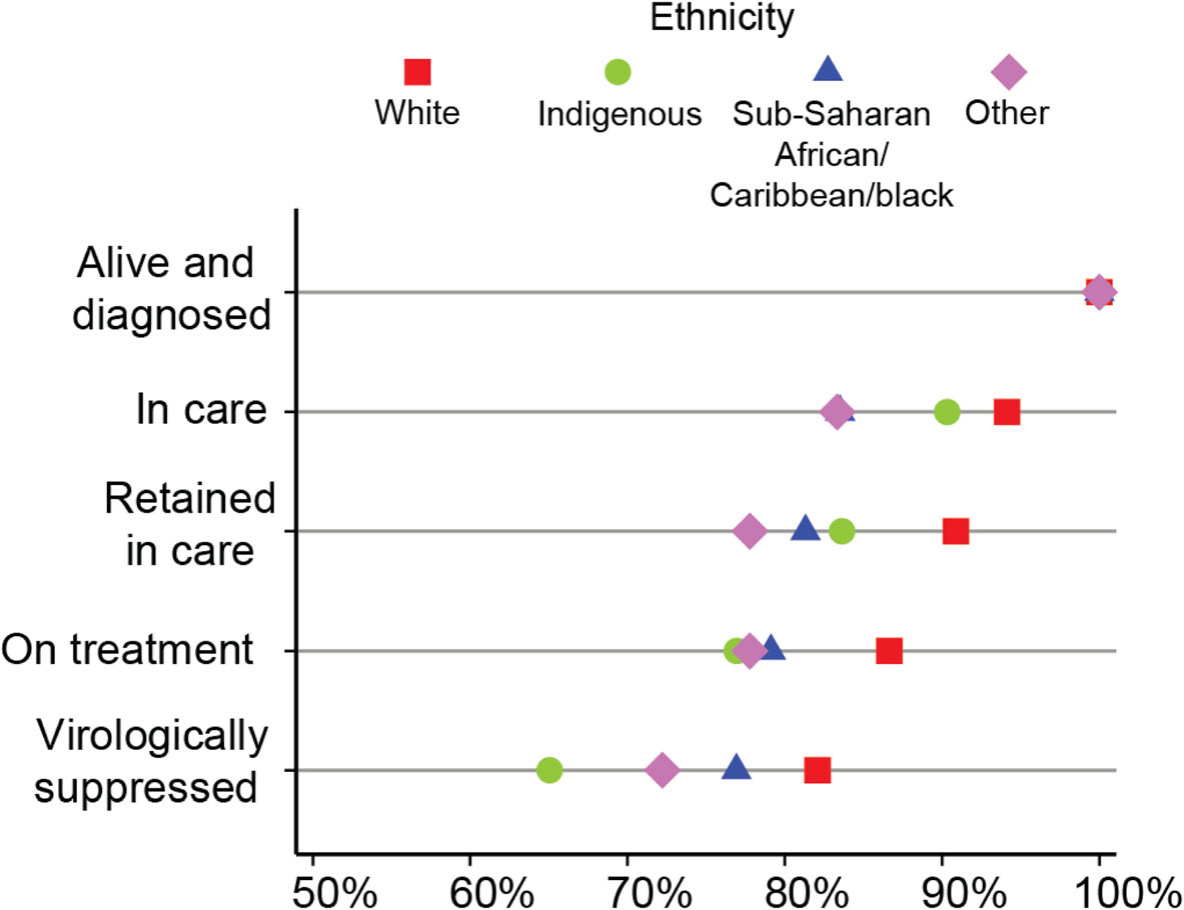
Inequalities across the Manitoban HIV care cascade, by ethnicity. *N* = 703 at *alive and diagnosed*

Compared to all other ethnicity categories white cohort participants comprise the greatest proportion in each step across the cascade (Fig. 5). The proportions of participants identifying as sub-Saharan African/Caribbean/black (ACB) and those in the Other ethnicity category are relatively low in the *in care* step (83.5 and 83.3%, respectively), whereas the proportions of Indigenous participants in the *on treatment* and *virologically suppressed* steps are relatively low (77.0 and 65.1%, respectively). Accordingly, Model 4 in Table 4 highlights that compared to white participants, the adjusted odds of being *in care* is significantly lower for ACB participants (AOR = 0.34, 95%CI:0.15–0.73) and being categorized as *retained in care* is less likely for both ACB (AOR = 0.43, 95%CI:0.22–0.86) and Indigenous (AOR = 0.57, 95%CI:0.33–0.97) participants. Furthermore, the odds of being *on treatment* and reaching the *virologically suppressed* step are approximately half for Indigenous participants compared to white participants (AOR = 0.55, 95%CI:0.33–0.92 and AOR = 0.54, 95%CI:0.34–0.84, respectively).

#### Immigration status

**Fig. 6:**
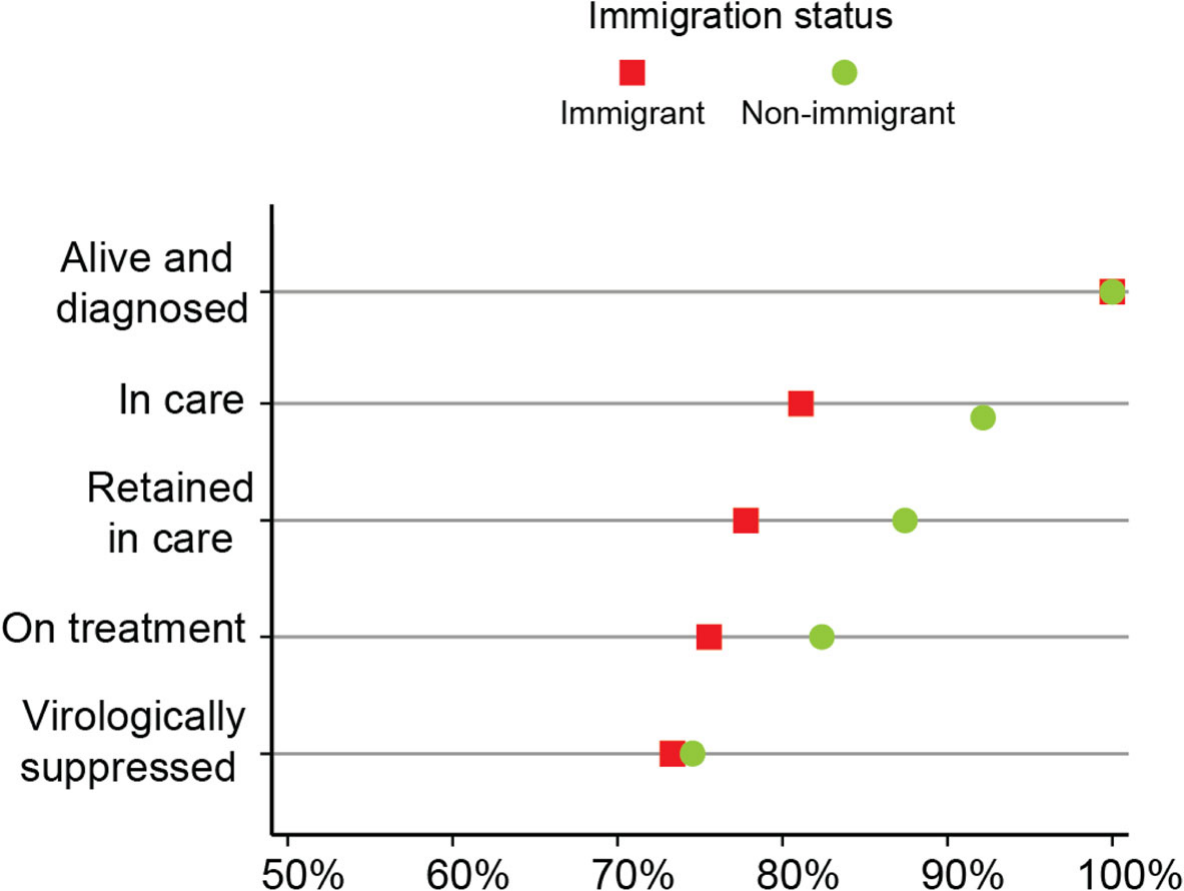
Inequalities across the Manitoba HIV care cascade, by immigration status. *N* = 703 at *alive and diagnosed*

After controlling for age group, sex, and ethnicity, a participant’s immigration status was not found to significantly influence their odds of being in a given cascade step (see Table 4, Model 5). However, the equiplot in Fig. 6 and data in Table 3 highlight a number of important inequalities between groups. In general, the proportion of participants who had immigrated to Canada in 2001 or later is notably lower than the proportion of non-immigrant participants in the *in care* (81.1% vs 92.2%, respectively), *retained in care* (77.8% vs 87.4%, respectively), and *on treatment* (75.6% vs 82.4%) cascade steps.

#### HIV exposure category

**Fig. 7:**
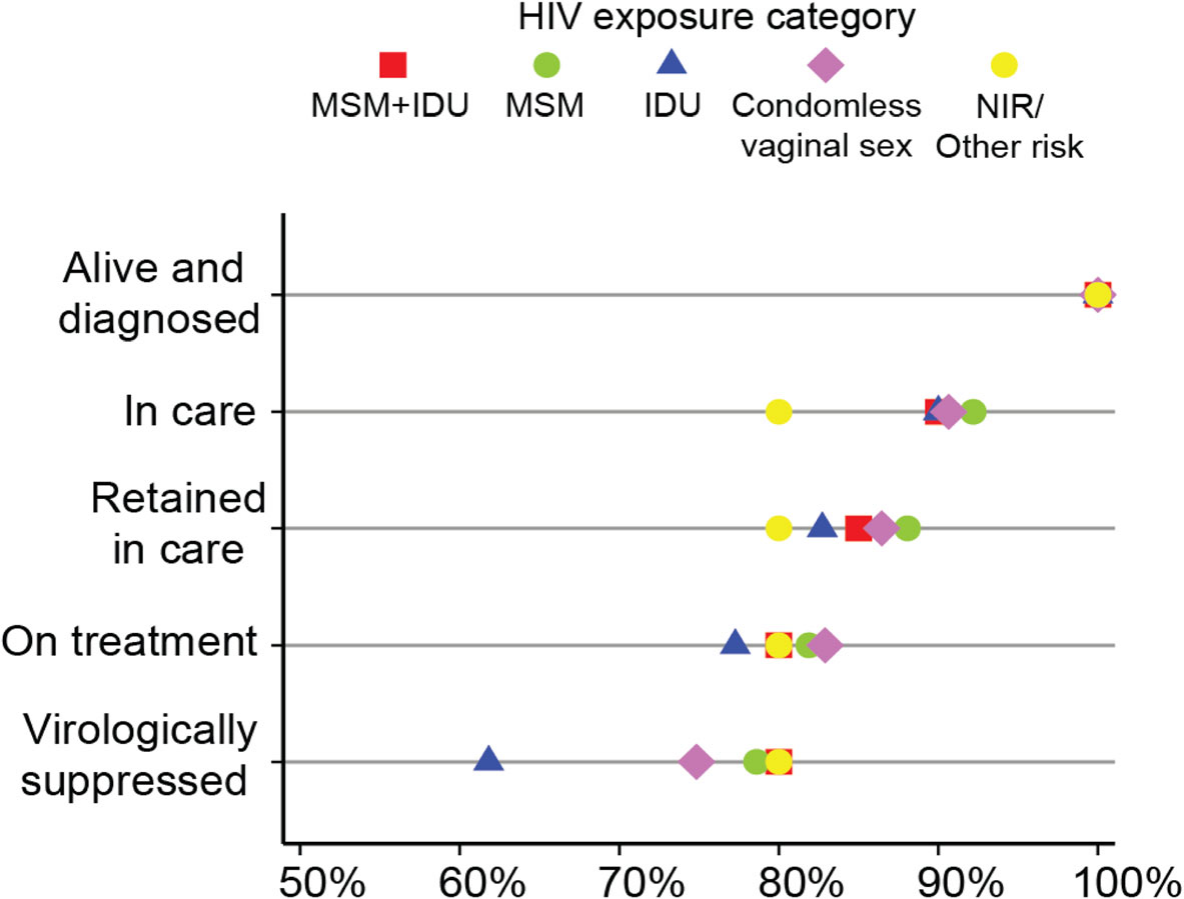
Inequalities across the Manitoban HIV care cascade, by HIV exposure category. *N* = 703 at *alive and diagnosed*. MSM, condomless anal sex between men; IDU, injection drug use; NIR, no identified risk

Disaggregating cascade data by HIV exposure category (Fig. 7) revealed few inequalities across cascade steps. Most notably, the proportion of participants whose primary HIV risk is injection drug use (IDU) categorized as *virologically suppressed* (61.8%) is at least 15% lower than the same for all other groups (Table 3). Compared to male participants reporting condomless anal sex with other men (MSM), participants with a history of IDU are half as likely to be included in the *virologically suppressed* step (Model 6 in Table 4, AOR = 0.48, 95%CI:0.27–0.85). The proportion of participants reporting condomless sex—either anal or vaginal—as their primary HIV exposure categories are distributed similarly across cascade steps (Fig. 7), although a slight inequality emerges between these groups at the *virologically suppressed* step (78.6% vs 74.8%, respectively).

## Discussion

In general, our data indicate that Manitobans living with HIV are progressing well along the cascade and are nearly meeting 90–90-90 targets—81.5% of those *alive and diagnosed* are on treatment and 91.3% of those *on treatment* are virologically suppressed (Fig. 1). However, equity analyses highlight important inequalities in progression along the HIV care cascade among cohort participants within different sociodemographic groups. Disaggregating our cascade data calls attention to clear inequalities in HIV care and outcomes by age, geography, and ethnicity among cohort participants. In general, individuals who are younger and non-white are less likely than their counterparts to reach subsequent cascade steps. Notable heterogeneity also exists along the cascade based on participants’ reported HIV exposure categories, with MSM progressing relatively well participants reporting IDU having relatively poor odds of reaching virologic suppression. These trends are not unprecedented; similar inequalities across the cascade have been noted in a variety of contexts [28–32]. While our multivariable logistic regression analyses highlight specific inequalities of statistical significance across the cascade, the use of equiplots to analyze disaggregated cascade data is an innovative and important method for identifying inequalities that, although not statistically significant, are highly relevant and should be considered during programmatic planning and design to address population-level inequities in HIV-related health outcomes. On the quest to generate evidence that can inform policy and program development aimed at minimizing health inequities, using cascade data, both aggregated and disaggregated, to identify inequalities in health outcomes and service access, delivery, and utilization is necessary, but insufficient. As Seckinelgin [6] and Zamora and colleagues [11] have argued, employing additional methodologies, such as qualitative inquiry and community-based participatory research, to inform policy and program design is crucial.

When it was first introduced in 2011 by Gardner and colleagues [4], the spectrum of engagement of HIV care, which ultimately became the HIV care cascade, was framed as an analytic tool for mapping individual- and population-level progression through the continuum of HIV care services. Specifically, Gardner’s model [4] provides a framework through which to determine the proportion of individuals in various stages along the continuum, and to “explore the potential impact of interventions to improve engagement in care” (p.795). However, over time, the cascade, and its simplified counterparts, the 90–90-90 Initiative [7] and the 95–95-95 Fast-Track targets [33], have been adopted or endorsed by global technical and policy normative bodies (e.g. UNAIDS [7, 33] and the World Health Organization [34]), and used to guide and influence international HIV policy development [6]. Expanding the utility of the cascade framework from an analytic tool to a large-scale decision- and policy-making framework is problematic because, as Seckinelgin [6] notes, “the model itself does not analyse the broader socio-political and economic conditions that interact with individuals’ experiences of HIV and that inform their decisions to engage with health services” [6]. In the process of developing health policies that align with the principles of health equity and the SDG commitment to leaving no one behind, it is essential for decision-makers to thoroughly consider how social determinants of health influence and manifest inequalities in health outcomes and access to health services [10, 35].

As we demonstrated, performing equity analyses using HIV care cascade data, and illustrating inequalities along the cascade using equiplots, concisely draws attention to points along the cascade at which specific groups of individuals are unable to optimally engage in HIV care or reach target health outcomes. Still, these analyses provide insufficient context or explanation for leakages along the cascade. In order to appreciate nuances in observed inequalities across the cascade, and to understand, for example, why people are having a hard time engaging in their HIV care and how to best support equitable access for all, complementary research approaches, namely qualitative inquiry and community-based participatory research, are necessary [6, 11, 36].

Next, using our analyses in this paper as an example, we demonstrate one way to expand the utility of innovative data visualization techniques, such as the equiplot. In Fig. 4, obvious discrepancies exist in the proportions of cohort participants from different geographic regions in Manitoba falling within each cascade step. Of particular interest to the Manitoba HIV Program may be the relatively low proportions of individuals living in western Manitoba categorized as *in care* and the substantial leakage at the *virologically suppressed* step among participants living in southern Manitoba. Indeed, previous research has also identified substantial geographic heterogeneity in engagement in HIV care [31, 37], some of which may be attributable to limited access to services due to physical distance [38], or other individual-, community-, and structural-level barriers [32, 37, 39]. Although our disaggregated analyses have provided a useful starting point for understanding geographic heterogeneity in HIV care in Manitoba, further mixed methods explorations will be necessary to delve into understanding the complex circumstances that shape inequities along the cascade before meaningful recommendations can be made to inform local programming or policy. A next step to understand geographic inequalities will require further disaggregating data (e.g. by sex, age, socioeconomic status) to uncover whether specific groups *within* geographic regions are further vulnerable to suboptimal engagement. Once a reasonable level of granularity is achieved in identifying “key groups” who may require additional support to engage in HIV care, program adjustments and policy development should then be based upon further-contextualized understandings of barriers to engagement in care through meaningful community involvement in program and policy decisions [11, 36]. Policy and programmatic decisions aimed at reducing health inequities must incorporate nuanced conceptualizations of how the various factors that influence access to and engagement with necessary and appropriate health services interact and overlap [39] to create specific conditions that prevent individuals from progressing through the HIV care cascade and other care continua [6]. If these complexities are not considered, and instead the linear logic inherent to the cascade [6] is privileged, policies intended to reduce gaps in equity will continue to miss the mark.

### Study limitations

This study has a few limitations that must be noted. First, a number of limitations inherent to the design of our clinical cohort have been described in detail elsewhere [14–17]. Opportunities to participate in the cohort are introduced to individuals in the context of their clinic appointments with the Manitoba HIV Program; participation is optional and does not impact the way that HIV care and other services are received. Still, we have to assume that selection bias may be influencing our analyses, and actual engagement in HIV care among the clinic population may be lower than we are able to assess from the cohort. For the same reasons, we cannot presume that our findings are generalizable to the broader population of people living with HIV in Manitoba, although previous work suggests that these data are reasonably representative of larger population in HIV care in Manitoba [15]. Second, using the LHIV-Manitoba cohort as a starting point for the first step of the cascade means that we cannot ascertain information about the proportion of people living with undiagnosed HIV in Manitoba and thus limits our ability to generate provincial estimates for all 90–90-90 targets. Finally, available data were limited such that we were unable to analyse the Manitoban HIV care cascade by income, level of education, or other socioeconomic status (SES) indicators. This will be an important addition to this work, which we will undertake as we move forward with more detailed analyses of our cohort data.

## Conclusion

Our findings highlight a need for further investigation into the complex and dynamic circumstances that shape the lives of people living with HIV in Manitoba and, ultimately, influence the ability of certain groups to engage in their HIV care. While our cascade equity analyses provide a useful starting point to work toward achieving health equity and leaving no one behind for people living with HIV in Manitoba, eliciting meaningful policy and programming change will require deeper, more comprehensive work to understand barriers and facilitators to engagement in care.

## Data Availability

Researchers interested in accessing data related to the LHIV-Manitoba cohort should be directed to Dr. Marissa Becker (marissa.becker@umanitoba.ca). All access requests for individual-level data must be accompanied by proposals for the research projects and will be subject to approvals by Health Research Ethics Board at the University of Manitoba, as well as the researchers’ home institution. Aggregate and/or de-identified data may be shared with fewer restrictions pending review by the LHIV-Manitoba study team.

## Abbreviations

95%CI: 95% confidence interval
ACB: Sub-Saharan African/Caribbean/Black
AOR: Adjusted odds ratio
CIHR: Canadian Institutes of Health Research
HIV: Human immunodeficiency virus
ICD-9/10: International Classifications of Diseases-9/10
IDU: Injection drug use
LHIV: The *Advancing Primary Healthcare for Persons Living with HIV in Canada* Study
MDGs: Millennium Development Goals
MHSAL: Manitoba Health, Seniors and Active Living
MSM: Men having condomless anal sex with other men
PHAC: Public Health Agency of Canada
SDGs: Sustainable Development Goals
SES: Socioeconomic status
UM-HREB: University of Manitoba Health Research Ethics Board
